# The sustainability of public health programs following donor transition: A comparative case study of HIV services and maternal and newborn care in Uganda

**DOI:** 10.1371/journal.pone.0341328

**Published:** 2026-02-02

**Authors:** Henry Zakumumpa, Eric Ssegujja, Marjorie Kyomuhendo, Beryl Maritim, Timothy Musila, Freddie Ssengooba

**Affiliations:** 1 Makerere University, School of Public Health, Kampala, Uganda; 2 Makerere University, Directorate of Research and Graduate Training, Kampala, Uganda; 3 Makerere University, College of Humanities and Social Sciences, Kampala, Uganda; 4 KEMRI Wellcome Trust Research Programme, Nairobi, Kenya; 5 Republic of Uganda Ministry of Health, Kampala, Uganda; University of Nigeria - Enugu Campus, NIGERIA

## Abstract

Although there is emerging evidence on the impact of donor transition on health programs, there is little research comparing sustainability outcomes across health programs. We sought to compare drivers of health program sustainability concerning maternal and newborn care in Western Uganda after the end of the ‘Saving Mothers Giving Life’(SMGL) project and HIV services in eastern Uganda following loss of PEPFAR support.

**Methods:**

We report qualitative findings from a larger mixed-methods study. In-depth interviews were held with Ministry of Health officials (n = 11), district health teams (n = 27), facility in-charges (n = 39) and representatives of donor-implementing organizations (n = 22). Data were collected in eight districts in Western and Eastern Uganda. Data were analyzed by thematic approach based on the five themes proposed under the Integrated Sustainability Framework (ISF).

**Results:**

Our case studies identified several enablers and hindrances to the sustainment of public health gains across HIV and Maternal and Newborn Health (MNH). The recipient government appeared to assign a higher political priority to MNH relative to HIV following donor transition. MNH attracted multiple external funders after the end of SMGL support. In terms of donor transition processes, the MNH intervention was perceived as a ‘terminal’ project, while PEPFAR support was perceived as more ‘open-ended’. In contrast to districts in Eastern Uganda, which lost PEPFAR support, internal ‘program champions’ were identified in districts in Western Uganda. Differences in disease control approaches were identified; HIV was described as more ‘capital intensive’ with more ‘recurrent’ needs compared to MNH programming. The expanded MNH workforce (such as nurses and midwives) was transitioned to the public sector payroll, while PEPFAR-salaried officials were not. Participants perceived the SMGL project on MNH to have been more embedded in the local health system while PEPFAR support was perceived as more ‘vertical’.

**Conclusions:**

Our analysis suggests that variations in sustainability outcomes cross the two focus projects stem from differences in donor aid delivery mechanisms, transition processes and domestic political priorities. Our study suggests that donor transition is not a ‘one size fits all’ phenomenon regarding health programs, which has implications for planning for donor transition in Uganda and similar settings.

## Introduction

Over the past three decades, low- and middle-income countries (LMICs) have achieved remarkable public health gains with substantial development assistance for health (DAH) [1–3]. DAH takes various forms, ranging from bilateral support, such as the United States President’s Emergency Plan for AIDS Relief (PEPFAR), to multilateral mechanisms, such as Global Alliance for Vaccine Initiative (GAVI) and the Global Fund for AIDS, Malaria, and Tuberculosis (GFTAM) [4].

Several studies have demonstrated the long-term impact of DAH on population health, such as a reduction in HIV-associated mortality [2], maternal and newborn mortality [1], increased uptake of immunization [3], and malaria control [5].

The recent freezing of U.S. international assistance for global health and cuts in foreign aid by major western donors [6] coupled with the uncertainty occasioned by recent global geopolitical tensions, and the associated increased defense spending in traditional Western donor countries have placed the long-term sustainability of donor-supported health programs in LMICs into doubt [7].

Although DAH is of critical importance to LMICs, much of this external assistance is time-limited and delivered through short-term projects typically lasting two to five years [2]. Additionally, this support is often provided outside the national budget and channeled through stand-alone projects which could hinder long-term planning and have limitations in building lasting health system capacity [2]. It is common to find that when such funding ends, progress is often reversed, and recipient governments struggle to sustain health coverage after donor exit [8–10].

Despite the increasing frequency of donor transitions in health [6], there is limited research examining the impact of loss of this aid on long-term population health outcomes, such as coverage of health services after donor transition [6]. Proctor and colleagues [11] posit that there are distinct stages or ‘life cycle’ of interventions after they are implemented within organizations. These ‘implementation outcomes’ or stages include acceptability, adoption, feasibility and sustainability [11]. Extant implementation literature [11,12] has called for analyses aimed at understanding the long-term sustainability of donor-funded public health programs. While many studies have focused on the initial adoption, implementation and acceptability of donor-funded public health interventions [12] there has been comparatively less empirical attention on influences on the long-term sustainability of public health interventions after donor exit. This is a priority research gap, which our study addresses. This gap has been identified by several systematic reviews [13–15].

Although there is emerging evidence on the effects of loss of donor aid on health programs [8,9], several studies have focused on individual health programs such as malaria control [5] and immunization coverage [3]. There is little research comparing variability in sustainability outcomes across more than one health program, a void which this paper begins to fill.

Studies comparing cross-programmatic sustainability outcomes across diverse health programs or disease conditions [12] are central to health policy and planning in LMICs since they can amplify our understanding of whether loss of donor aid has similar effects across health programs. Likewise, these analyses are beneficial to recipient governments, external donors, health program planners and even the primary beneficiaries, as well as for Civil Society Organizations (CSOs) involved in advocacy for vulnerable populations in the context of donor transition [10]. Studies on donor transition can inform strategies for maintaining coverage of services after loss of donor funding [12].

### Uganda country context

Uganda is a low-income country according to the World Bank. The total budgetary allocation to health has been increasing over the years but spending per capita on health is steadily declining and currently stands at 7% which is below the minimum threshold of 15% agreed during the Abuja declaration [16,17]. The rapid growth in population needs at 3% has culminated into coverage gaps, slow progress and quality issues. Out-of-pocket expenditure accounts for one of the highest health expenditure (43%) in Uganda [16,17]. In total, 96% of private health services are paid for by households. The 16% public finance contribution to health spending remains too low to offer financial protection for the over 44 million Ugandans [16,17]. The country heavily relies on donor funding with the national health accounts reflecting up to 42% of health budget funded with donor support in 2015/16 of which 30% was provided outside the national budget. Other sources include the private sector which contributes 2% of the annual health expenditure.

The country is heavily dependent on multi-lateral and bilateral donors for health care financing. However, for selected health programs such as the national HIV response, external assistance accounts for as much as 83% of HIV spending [18].

Due to sustainability concerns, plans are underway to decrease health spending by households through raising domestic resources for health despite the limited fiscal space albeit strong political will.

### The two DAH interventions

**Fig 1:**
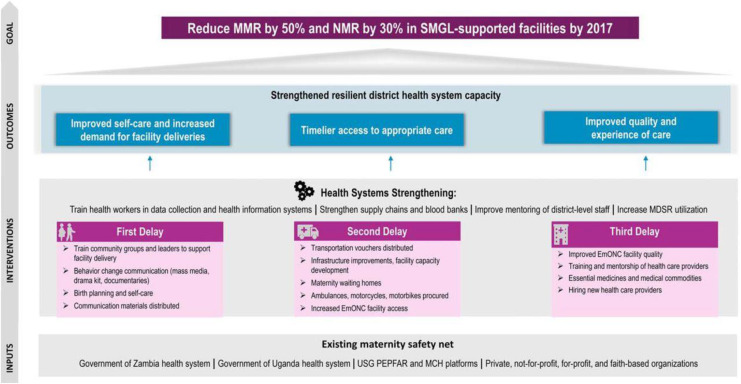
The SMGL Intervention design ‘theory of change’.

Between 2012 and 2016, the United States Agency for International Development (USAID), through the ‘Saving Mothers Giving Life’ (SMGL) project, launched a five-year intervention aimed at reducing maternal and newborn mortality in four high-burden districts in Western Uganda (Kyenjojo, Kabarole, Kamwenge and Kyenjojo) serving a broader population of more than 1.2 million Ugandans [1]. **Fig 1** shows that the SMGL intervention aimed at reducing maternal mortality by 50% by addressing the ‘three delays’, namely, (i) decision to seek care, (ii) timely access to health care, and (iii) quality healthcare [1]. The SMGL initiative’s health system strengthening approach included expanding the workforce for delivering maternity services, supporting physical infrastructure such as expanding maternity wards and building new hospital laboratories, providing ambulances to district and sub-district hospitals, and transporting vouchers to expectant mothers through private sector engagements [1]. SMGL targeted more than 263 (out of 456) facilities in mid-Western Uganda that provide comprehensive emergency obstetric care. **Fig 1** shows the three groups of factors which may stop women from accessing maternal care.

Uganda has 1.4 million people living with HIV, one of the highest HIV burdens in Eastern and Southern Africa, the epicenter of the global HIV epidemic [19]. From 2004−2015, Eastern Uganda benefited from PEPFAR support in providing comprehensive HIV services such as increasing awareness of and expanding access to antiretroviral therapy (ART) at routine points of care country-wide, increasing HIV testing and scaling up HIV prevention interventions such as Voluntary Medical Male Circumcision (VMMC) and distribution of condoms. PEPFAR support followed a public health approach to HIV epidemic control through a generalized national HIV response countrywide. Between 2015 and 2017, PEPFAR ceased support to ten ‘low burden’ districts in Northern and Eastern Uganda following a new strategy of aligning aid with disease burden at sub-national level. The process of transition was gradual and involved protracted engagements with national-level stakeholders [20,21]. The impetus for pivoting to regions with a higher disease burden at sub-national level as opposed to a generalized national HIV response was determined by PEPFAR at a global level through a strategic vision dubbed ‘PEPFAR 3.0’ [21]. The ‘geographic prioritization’ (GP) policy was endorsed by the Office of the Global AIDS Coordinator, which is responsible for setting policy for PEPFAR-supported countries globally. The newly unveiled vision for PEPFAR for the period 2013–2019 signaled a new strategic thrust towards improving the ‘allocative efficiency’ of PEPFAR aid in the quest to achieve UNAIDS’ 90-90-90 targets in its 15 ‘focus countries’ following more than a decade of ‘emergency aid’ in countries with a high HIV burden [21].

The objective of this study is to compare drivers of health program sustainability with respect to maternal and newborn care in Western Uganda after the SMGL project and HIV services in Eastern Uganda following loss of PEPFAR support. The study objective was explored utilizing five deductive themes (inner context, outer context, characteristics of the intervention, processes and intervention characteristics) [11].

## Materials and methods

### Research design

This qualitative study was part of a larger mixed-methods study examining the impact of donor transition on health service coverage and health systems in Uganda [22]. The quantitative component entailed a trend analysis of selected maternal and newborn health indicators as well as HIV services based on secondary analysis of routinely collected data in the District Health Information System or DHIS-2. The study was part of a multi-country study in six countries in LMICs, including Uganda [23]. This study sought to understand the impact of cessation of external assistance on health coverage and health systems in LMICs [23].

In this qualitative paper, we focus on the perceptions of local stakeholders in Uganda, such as district health teams and frontline health workers, on the impact of the loss of donor support on two focus health programs: HIV services in Eastern Uganda and maternal and newborn care in Western Uganda.

Uganda was selected as a country case study because it offered us a unique case of having hosted two distinct multi-million-dollar public health projects at sub-national level with clearly defined donor aid packages, defined timelines for implementation and well spelt out public health goals (such as achieving a 50% reduction in maternal deaths) [1].

### Theoretical orientation

This paper is informed by conceptual papers on the subject of the sustainability of public health interventions [13–15]. This is with respect to the elements that influence program sustainability following the end of project funding [11,12]. Within the field of implementation science, sustainability is conceptualized as a later stage in the continuum of the overall ‘life cycle’ of an intervention [12]. The initial implementation stages include adoption, fidelity, feasibility, acceptability and effectiveness [12]. Sustainability is “the continued delivery of an innovation or intervention, potentially after adaptation, at a sufficient level to ensure the continued health impact and benefits of the intervention” [24]. The review articles by Stirman and colleagues [25], Scheirer and Dearing [26], Shediac-Rizkallah and Bone [27] informed this study. More specifically, we adopted a multi-level analytical framework proposed by Shelton and colleagues under the Integrated Sustainability Framework (IFS) [11]. The later framework proposes five themes; a) outer context factors, b) inner context factors, c) characteristics of the intervention d) ‘processes and e), intervention characteristics [11]. We selected the ISF framework because it is informed by a rigorous review of the literature on factors influencing health program sustainability [11]. These five themes in the ISF framework remain consistent in more recent reviews of the literature on public health program sustainability [13–15].

We utilized the ISF framework in three ways. We utilized the five ‘sub domains’ proposed in the ISF framework in constructing our in-depth interview guides. Secondly, we broadly used the ISF framework as an overarching deductive framework for our qualitative data analysis. Thirdly, we used the ISF framework in the categorization and presentation of findings which we sectioned according to the five deductive ‘sub domains’ (e.g., inner context, outer context factors).

### Study sites and sample selection

Uganda runs a decentralized health service delivery system [28], where the mandate for social services provision has been devolved to sub-national units known as districts. Hence, districts were the primary unit of study.

With respect to maternal and newborn care, we selected three districts, Kamwenge, Kyenjonjo, and Kabarole, in Western Uganda, that benefitted from USAID’s Saving Mothers Giving Life (SMGL) support in reducing maternal and newborn mortality between 2012 and 2016 [29].

Regarding PEPFAR support, we selected two districts, Luuka and Bulambuli, in Eastern Uganda which lost PEPFAR support between 2015 and 2017. Within these two districts, 241 health facilities lost support in HIV services delivery following the implementation of the ‘geographic prioritization’ policy. This entailed a shift by PEPFAR away from a generalized national HIV response to a pivot towards increasing aid, where the HIV burden was highest at the sub-national level in Uganda. Consequently, there was a reduction or cessation of funding of support in districts in Uganda with a ‘low HIV burden’ [22].

Table 1 indicates the donor package for the two SMGL and PEPFAR projects in the Western Uganda and Eastern Uganda case study districts, respectively.

**Table 1:** A comparison of donor aid packages across HIV and MNH interventions.

	SMGL/MNH	PEPFAR/HIV
Workforce expansion	• Midwives, Nurses, medical doctors	• Data managers, counsellors, supply chain experts
Medicines and commodities	• Provision of medical oxygen cylinders	• Antiretrovirals, condoms
Information systems	• MNH quality improvement metrics	• Hiring data clerks at facilities
Laboratory infrastructure	• New health facility laboratories	• Regional lab hubs, viral load testing
Community outreach	• Motorcycle taxi vouchers and ambulances	• Community linkage facilitators, mobile HIV testing
Sub-national aid governance arrangements	• Baylor Uganda as an implementing organization	• STAR-E (Strengthening TB and HIV & AIDS Responses in Eastern Uganda Project) • RHITES-E (Regional Health Integration to Enhance Services in East-Central Uganda)
Primary beneficiaries	• Expectant mothers attending 263 facilities.	• People with HIV attending 241 facilities.
Implementing organization	• Baylor Uganda ($ 200 million for Uganda and Zambia)	• STAR-E/ RHITES-E ($65 million program)

### Data collection

Data were collected with a multi-level lens in mind. We utilized purposive sampling to enroll a diverse set of participants in the study. We purposively sampled participants from the two case-study projects of HIV and MNH services. We sought a multi-level understanding of drivers of health program sustainability and hence sought a broad range of participants ranging from national-level actors, sub-national level officials to facility-level personnel giving us a total of 98 participants. We engaged national-level actors, including Ministry of Health officials and representatives of PEPFAR/USAID at the national and sub-national levels, comprising district health teams (DHTs). The other study participants consulted were representatives of PEPFAR/USAID at the regional and facility levels, comprising health personnel in charge of HIV clinics and midwives in maternity wards. Data were collected between 15th January to 30^th^ May 2022. Participants were paid a modest time compensation of Uganda shillings 20,000 (USD$ 5.7).

**Table 2** shows the category of participants across the case-study health programs.

**Table 2:** Number of study participants across case-study health programs.

	HIV/PEPFAR	MNH/USAID
Ministry of Health officials	04	07
District Health Teams	18	09
Representatives of donor agencies	16	06
Health workers	25	14
	**62**	**36**

#### In-depth interviews (IDIs).

We utilized a pre-tested interview guide which was sectioned according to the five overarching themes proposed in the Integrated Sustainability Framework (e.g., inner context and outer context factors) [11]. The face-to-face interviews were conducted by the first and second authors in the offices of participants. Two Research Assistants took notes during the proceedings of the interviews and operated the recorder. The interviews were conducted in English and on average, lasted between 45 and 60 minutes. The interviews were audio-recorded with the consent of participants.

#### Desk reviews.

Case studies utilize multiple data collection sources [30,31]. We adopted a triangulated approach to supplement our predominant primary data collection through in-depth interviews. We reviewed grey literature sources such as published reports from PEPFAR, SMGL and implementing organizations or partners (IPs) with respect to 2015–2020 with respect to HIV services and 2016–2020 for MNH services [32,33]. We also examined the websites of implementing partners documenting their intervention design, such as that of STAR-E (Strengthening TB and HIV & AIDS Responses in Eastern Uganda Project), the implementing partner for Bulambuli District in Eastern Uganda [34]. We also reviewed evaluation reports of SMGL with respect to beneficiary districts in Western Uganda [35]

The inclusion criteria was such that we included the published and grey literature with respect to the two projects (such as end-line evaluation reports). We excluded opinion pieces, commentaries and editorials.

### Ethical clearance

This study received ethical approval from Makerere University School of Public Health’s Institutional Review Board under instrument: SPH 2021−128 and from the Uganda National Council for Science and Technology under instrument: HS2112ES. All interview participants signed a written informed consent form before participating in the study. Our engagement with human subjects in this study strictly followed international guidelines such as the Declaration of Helsinki.

### Data analysis

We followed qualitative data analysis approaches recommended by Miles & Huberman (1994) [36]. More specifically, we used the framework approach to qualitative data analysis [37].

The audio files generated from in-depth interviews were transcribed verbatim into text transcripts. Transcripts were then uploaded into qualitative data analysis program (ATLAS-ti Center, Berlin) for storing and organizing our qualitative data. Broadly, data were analyzed in four stages although this was a largely an iterative process. In the first stage, two authors (HZ, ES) read the transcripts multiple times for data familiarization [37]. In the second stage, four authors (HZ, ES, MK, BM) devised an inductive coding scheme from the first few interview transcripts in a bid to identify influences on health program sustainability across the two projects (e.g., the presence of internal program ‘champions’, availability of successor external funding, absorption of project staff onto the government payroll). The resulting coding scheme was applied to all subsequent transcripts. Two authors (HZ, ES) ensured that inter-coder reliability was achieved by the different coders and that they applied the same codes to transcripts in a fairly consistent manner. Three authors (HZ, ED, MK) categorized the inductively-derived codes and grouped them into categories under the five deductive themes (inner context, outer context, transition processes, intervention characteristics, and implementer characteristics) as proposed by Shelton and colleagues in the Integrated Sustainability Framework [11]. Hence we utilized a hybrid approach of inductive and deductive coding and theme development [38]. The resulting themes were compared across the two projects in a team-based process. Disagreements in theme development were resolved through consensus. The fourth step involved overall data synthesis and interpretation by all authors. These were later presented during a validation meeting with study participants who provided their input to the emergent findings.

## Results

The findings emerging from the two case studies are presented in two sections. Section A focuses on the perceived health program sustainability outcomes post-transition across the two projects. Section B explores the drivers of program sustainability based on the five deductive themes [12] across the two case-study projects.

### Section A: Perceived health program sustainability outcomes

Interviews with participants involved in the implementation of both interventions revealed that the two case-study projects offer contrasting post-transition sustainability outcomes. For the SMGL or the MNH intervention, qualitative data suggest that in the intervention districts in western Uganda, there were sustained reductions in maternal and newborn deaths and a sustained increase in the number of births occurring at facilities between 2017 and 2019 when compared to 2012–2016 during USAID project funding. District health teams pointed to data in the district health information system (DHIS-2), which shows reductions in maternal and newborn deaths in all but one of the districts -six years after end of the SMGL project in 2016 [29].

*‘What the data is telling us is that in this district, between 2016 and 2019, reductions in maternal and neonatal mortality and increased facility deliveries were sustained and even exceeded post-transition’* [District health team, Western Uganda, 03]

On the other hand, there was consensus across district health teams and facility-level participants in districts in Eastern Uganda which lost PEPFAR support that there was a reversal in HIV epidemic control post-transition (2017–2021) and that the districts hadn’t yet regained their pre-transition disease control for the period between 2012 and 2015 when they still had PEPFAR support. Participants indicated that during PEPFAR transition the districts which lost support were indicated as having an HIV prevalence of 1% in 2015 and that a national HIV prevalence study of 2020 study suggested that HIV prevalence had risen to 2% [22]. A recurring theme across Bulambuli and Luuka districts was perceptions of declining HIV epidemic control manifested in multiple ways, such as declining viral load suppression and community transmission of HIV.

*‘The lagged impact of the loss of PEPFAR funding in HIV programming in the district is that we are doing very badly in achieving viral load suppression rates. We are failing to achieve the global target for viral load suppression. Viral load is too high in the population, which means that (HIV) transmission is too high. We urgently need to bring down the viral load in the community* [District health team, Eastern Uganda, 06]

Similar sentiments on the hurdles in sustaining gains in HIV epidemic post-transition in previously PEPFAR-supported districts abound.

*‘In 2015, STAR-EC (PEPFAR IP) exited. We were meant to transition to full support by the Ministry of Health, but this did not materialize. Other districts in the same region continued receiving PEPFAR support for HIV interventions. So, this transition greatly affected us. Most of the interventions that were being supported to ensure that clients were offered quality HIV care and that they were being retained in (HIV) care ceased. For retention of clients in the care, you have to do a follow-up right up with their households, but that was all donor-funded. The district could not afford to go and follow up on these clients. Problems emerged around viral load monitoring, and issues of TB contact tracing became a problem because PEPFAR was also supporting them. Without a doubt, we suffered setbacks in HIV control in this district’* [District health team, Eastern Uganda,02]

Participants from Luuka and Bulambuli Districts were consistent in relaying the notion that loss of PEPFAR support contributed to a reversal in gains in HIV epidemic control in their districts when compared to the pre-transition phase. District health teams pointed to findings from a national HIV survey, the Uganda Population-based HIV Impact Assessment (UPHIA) 2016–2017, published in November 2019. The survey shows that both Luuka and Bulambuli districts had an HIV prevalence of 2.2% at the end of 2020, which contrasts with the HIV prevalence of 0.64% in 2015 prior to transition [21].

*‘When Luuka District was being transitioned from PEPFAR support in 2015 they said our HIV prevalence was at less than 1%. The latest national HIV prevalence survey released in 2020 shows that our HIV prevalence as a district stands at 2.2%. The transition was a mistake*’ [District health team, Eastern Uganda, 01]

### Section B: An exploration of drivers of program sustainability

The findings are categorized under the five deductive themes proposed under the Integrated Sustainability Framework (IFS) [12].

#### Outer context.

**Alternative external assistance:** There were observed differences in the ability to attract alternative external assistance after the end of project funding across the two projects. In the case of maternal and newborn care, district health teams in the three SMGL-supported districts in Western Uganda indicated that they attracted several new funders after the end of SMGL project funding. A World Bank-funded intervention known as Uganda Reproductive, Maternal and Child Health Services Improvement Project (URMCHIP) was implemented between 2017 and 2023 in case study districts with program targets that mirrored those of the SMGL project. In addition, Belgian support through the ENABEL program was launched in 2017, introducing a results-based financing intervention for improving MNH services in the region. ENABEL offered financial incentives for outcome indicators directly aligned with the SMGL project goals. For instance, financial incentives were provided directly to the health workers if their facilities attained MNH targets such as reduced maternal mortality.

*‘When ENABEL came in I think people worked hard because it also had that component of giving incentives. They said that 40% of the proceeds from results-based financing would go directly to staff as an incentive. So, it motivated the staff to work hard without them being pushed. There is a way they have been looking for clients because they need numbers. They will tell you we need mothers to come for antenatal care early’* [Facility in-charge, sub-district facility, Western Uganda,04].

With respect to MNH causes in former SMGL-supported districts, additional external support materialized from other funders. Save the Children and the United Nations Children’s Fund (UNICEF) were reported to have provided project funding to improve the quality of maternal and newborn care in the region following the end of SMGL funding.

*‘We had new partners coming on board such as Save the Children. They came in when there was a very big gap in staffing in facilities in refugee hosting communities in the district. We told them these facilities need midwives. There is too much workload, but we don’t have money for recruiting midwives. We don’t even have a budget for new salaries. Then Save the Children said ‘We can recruit for you and facilitate you’* [District Health Team, western Uganda, 06]

Conversely, district health teams that lost PEPFAR support in Eastern Uganda indicated that no alternative external funders stepped in to fill the void left by PEPFAR transition. Interviews with district health teams in case study districts in Eastern Uganda revealed a vacuum in funding for HIV programming.

*‘When PEPFAR transitioned us from their support we were abandoned. Even the Ministry of Health did not do the basics like on-site support supervision. Between 2015 and 2020, I do not recall ministry officials setting foot in our HIV clinic. And our district local government is always hard up financially so even the district did not come to our rescue. We were left on our own’* [Facility in-charge, Eastern Uganda, 08]

#### Inner context.

**SMGL-recruited workforce transitioned to Uganda government payroll:** There was aggressive recruitment of SMGL project-salaried staff onto the payroll of district local governments to expand the MNH workforce in case study districts in Western Uganda. Priority was first given to key cadres crucial in delivery of MNH (such as midwives and nurses) and later to boosting their numbers in order to meet the renewed demand from patients due to enhanced service delivery capacity. The absorption of a large section of the SMGL-recruited workforce was frequently cited as a contributor to the sustainment of MNH indicators post-transition.

*‘We absorbed several former USAID staff because the salaries had been harmonized. For example, the project staff under Baylor Uganda (USAID implementer) are getting the same salary scales as other local government personnel. So, they were quickly absorbed. They wanted to join public service because it has more permanent terms of service’* [Facility in-charge, western Uganda,04].

In addition to expanding the maternity workforce, human resources for health support extended to mentorship support in routine service delivery. The use of ‘coaches’ and ‘mentors’ to build the necessary capacities among in-service health workers was an integral part of SMGL. A USAID-salaried workforce was deployed to fill staffing gaps across mid-western Uganda focus districts.

*‘They (funders) saw that the theatres were fully functioning, and maternity units were working, but our staff needed skills enhancement. So, mentorships and coaching came in…that is when you could see managers from Mulago (National Referral) Hospital coming here to the hospitals and sub-district facilities to do on-site mentorship so that our people get the skills. That was the time I could see consultant pediatricians coming here on-site’* [Facility in-charge, western Uganda,09]

On the other hand, districts in Eastern Uganda which lost PEPFAR support indicated that they lost PEPFAR-salaried workers. Although most of the workforce in HIV clinics across Uganda, especially mid cadres such as clinical officers and nurses, are on the Uganda government payroll, personnel gaps abound. Due to severe staffing shortages, which are common at busy HIV clinics, a select number of patients known as ‘expert patients’ are co-opted as informal staff of HIV clinics and are paid regular monetary allowances from funds provided by PEPFAR.

Owing to stringent annual HIV epidemic control targets set by PEPFAR, additional personnel are often recruited to augment the existing workforce at the national and sub-national level, such as HIV commodities supply chain experts. At the facility level, PEPFAR usually provides salary support for data management personnel, counsellors, and select cadres who are not provided for in the Ministry of Health establishment structure or the Public Service in general. At health facilities, data entry clerks, counsellors and community-linkage facilitators who depended on PEPFAR for regular monetary allowances for their roles at facilities lost this income.

After transition, districts in Eastern Uganda that lost PEPFAR support reported losing these PEPFAR-salaried workers. This loss of financial support impacted the operations of HIV clinics and their ability to meet staffing needs for routine service delivery.

*‘From the get-go, PEPFAR realized that it is practically impossible for civil servants on Uganda government payroll to deliver their ambitious HIV control targets. They came up with a strategy of supplementary manpower of diverse cadres such as lay workers, counsellors, data clerks, and TB contact tracers. So, each facility has such a team, from Health Centre IVs to Health Centre IIs’* [District health team, Eastern Uganda, 05]

Workforce losses were further compounded by the departure of sub-national managers responsible for planning and managing annual HIV epidemic control targets following the PEPFAR transition. For instance, to reduce chronic commodity stock-outs, PEPFAR hired supply chain experts to redistribute stock across the regions to ensure all facilities had adequate stock. For epidemic control planning, PEPFAR hired epidemiologists with advanced degrees to track trends in HIV indicators as part of routine HIV programming and planning. However, these cadres, typically with advanced degrees, are not provided for in current staffing structures of district local governments. Efforts to reform the establishment structure of districts have been met with wage bill budget ceiling limitations.

*‘Unfortunately, human resources are very expensive in terms of the cost of new recruitments. We have pleaded with the Ministry of Health to review our staffing norms without success because they tell us there is no money to pay for an expanded workforce. I can tell you, for example, that for a Health Centre III, we no longer need 19 people, but we now need 30 personnel, but the Ministry (of Health) is adamant’* [District health team, Eastern Uganda, 01]

Local government staffing structures did not provide the additional layer of subnational-level HIV programming cadre. To compound matters, the salaries paid to these subnational-level HIV program managers are several times higher than those paid to their peers on the public sector payroll. Most of these sought alternative employment in districts in Uganda that retained PEPFAR support.

### Increased co-financing by the recipient government

Our findings suggest that the SMGL intervention catalyzed increased Government of Uganda’s (GoU) responsiveness to MNH causes, especially at the subnational level. The fiscal space for MNH services delivery increased. Besides workforce expansion, there was increased domestic financial support for items such as maintenance of ambulances for expectant mothers compared to the pre-SMGL intervention.

Upgrading SMGL-supported facilities to a higher level of service delivery stood out as a distinguishing feature across the two projects. Several facilities were upgraded to a higher level of service delivery owing to SMGL investments in expanding MNH services infrastructure. This implied larger budget allocation from the recipient government, additional personnel commitments and more MNH commodity supplies. Consequently, facilities such as Rukunyu Health Centre IV in Kamwenge District were upgraded to the status of a general hospital after benefitting substantially from SMGL support.

*‘When SMGL came, we were a Health Centre IV. By the time they left, we had been upgraded to the level of a general hospital. They even constructed and equipped a maternity ward. They recruited about 15 midwives for us, including doctors, and trained them to work in the NICU, and these were all absorbed and are still within.*’ [Facility in-charge, western Uganda,11]

District health teams in western Uganda relayed the notion that the national government accorded MNH a higher political priority due to the issue of maternal deaths’ being a touchy subject to Uganda’s political class. Reports of maternal deaths at local facilities quickly reach local members of parliament and district political leaders. In a cultural context of extensive kinship ties, relatives of deceased expectant mothers are an important voting bloc that elected political actors can ill afford to ignore.

For districts in Eastern Uganda which lost PEPFAR support, district health teams and facility-level participants reported that after being transitioned from PEPFAR support in 2017, the funding gap in HIV programming occasioned by PEPFAR exit was not filled. It was indicated that the Ministry of Health and parent district local governments did not offer any substantive support to help affected districts cope with the enormous gap in funding for HIV programming in the post-transition years between 2017 and 2020.

### Presence of internal program champions

Regarding the SMGL districts, the presence of internal MNH ‘program champions’ at the subnational level was identified as a contributing factor to the sustainment of programmatic gains post-transition in Western Uganda. The level of commitment of the political and technical leadership of select intervention districts, such as Kamwenge District local government, stood out as outstanding.

*‘We had an excellent working relationship with political leaders in the district who make key decisions such as on budgets and recruitments. We asked them to approve the absorption of midwives on the payroll. So, even the political will is important, and the presence of goodwill by important decision-makers worked in our favour’* [District health team, Western Uganda,08].

Our key informant interviews revealed that these champions were instrumental in securing budgetary commitments from GoU such as committing the district wage bill towards absorbing SMGL-recruited midwives and nurses. In three SMGL districts internal champions were identified such as District Health Officers who went beyond the call of duty and were exceptionally committed to the MNH cause through securing in-puts such as commodities and medicines to facilitate service delivery by skillfully navigating GoU processes.

Conversely, interviews with district health teams and facility in-charges in Luuka and Bulambuli districts reported the absence of ‘program champions’ after losing PEPFAR support.

‘*When PEPFAR transitioned from us we were like orphans. I don’t remember any official from our district administration who came to our rescue. The Ministry of Health did not show up and frankly even at this facilitaty nobody came to our rescue. That is why I say we were like orphans’* [Facility in-charge, Eastern Uganda, 06]

### Perceived externalizing HIV funding challenges

District health teams in Luuka and Bulambuli contended that the GoU seemed to have an informal strategy of externalizing the challenge of funding the national HIV response to PEPFAR. Key Informants indicated that a donor-dependency culture had coalesced among local stakeholders in Uganda. Local stakeholders expected PEPFAR to change course and resume HIV funding.

*‘I think that the national government did not intervene to replace PEPFAR investments in HIV programming because it speculated that PEPFAR would make a comeback’* [Ministry of Health Official, 03]

Indeed, true to expectations, in October 2020, PEPFAR resumed support to Luuka and Bulambuli Districts after transitioning away from them between 2015 and 2017.

National-level informants reported that even locally based donor officials such as PEPFAR-Uganda technocrats were not wholeheartedly behind the strategy of pivoting away from ‘low burden’ districts, and they only got on board because, as stated by a key informant, ‘*the ship had sailed* [Ministry of Health Official, 05]. The impetus for pivoting to regions with a higher disease burden at the sub-national level as opposed to a generalized national HIV response was determined by the PEPFAR at a global level and not by locally based PEPFAR officials. Indeed, there was a subtle expectation that PEPFAR would change course.

On the other hand, the USAID-funded SMGL initiative was framed as a ‘terminal project’ to local beneficiaries from the outset. Both district health teams and facility-level participants consistently reported that the implementer made it clear from the outset that SMGL was a terminal project with no prospects for extension.

*‘I think USAID was consistent right from the start. They made it repeatedly clear that this was a five-year project. They kept drumming it into us. Even at the tail end, there were several meetings held with the district officials to mentally alert them that the project was ending’* [Facility in-charge, Western Uganda, 06]

### Implementer characteristics

#### Continuing support by SMGL Implementing partner.

There were contrasts in post-transition support by implementing agencies across the two projects. We learned from participants in Western Uganda, such as those from Kyenjonjo District, that Baylor Uganda, the regionally based USAID implementing organization for the SMGL project, continued offering financial support to the district hospital for MNH service delivery even after the end of the five-year project. This support was manifested in procuring expensive spare parts for hospital equipment acquired during the project, some of which were not available in-country and had to be sourced from outside Uganda. The continuing support extended to maintaining laboratory equipment and servicing ambulances for transporting expectant mothers from rural outposts.

*‘We were fortunate that Baylor kept helping us to maintain the equipment we received as part of the project even after the project had officially closed. They even imported spare parts from overseas, which we couldn’t find locally because these are very sophisticated equipment that not many hospitals in Uganda have’* [Facility in-charge, Western Uganda, 07]

As highlighted in an earlier quote from a district official about being ‘orphaned’ after the PEPFAR transition, district officials in Eastern Uganda indicated that the PEPFAR Implementing organization – RHITES-E stuck to the transition script.

### Intervention characteristics

#### Perceptions that HIV programming is capital-intensive.

Key Informant interviews revealed nuances in intervention approaches across the two case-study projects. PEPFAR officials strongly suggested that HIV epidemic control required a myriad of interventions at the population level. There was a widely held perception that comprehensive HIV prevention and treatment programs are relatively capital-intensive and that not many funders are willing to commit to the levels of investment needed to achieve epidemic control.

From the perspective of district health teams, to understand variations in sustainability outcomes across the two projects for HIV and MNH, one needs to appreciate the differences in programming needs and, therefore, financing needs from a disease control or epidemiological perspective.

District health teams in Eastern Uganda described HIV programming as relatively ‘capital intensive’. They perceived domestic financing sources as inadequate in meeting the multi-faceted demands of HIV epidemic control at the population level. There was a widely held perception that Uganda would continue to be dependent on external aid in its national HIV response.

‘*To me, the way I have seen HIV, if we are to sustain HIV epidemic control and continue offering quality HIV services, donor support is going to be necessary for a long time. This is because the Uganda government or Ministry of Health doesn’t have the kind of resources needed for HIV control at the population level. HIV is so demanding. It is not like other diseases or conditions. Without external funding, it is very difficult to retain these patients on treatment. Also, HIV disease management is multi-faceted because their (PWH’) illness affects them socially, economically, and physically. So, they need many interventions that necessitate donor support’* [District Health team, Eastern Uganda, 04].

The perceptions of district-level actors were that Uganda is heavily dependent on external donor aid for HIV relative to other health programs due to a range of reasons, including the sheer cost of HIV care and treatment such as procurements of antiretrovirals, viral load and other laboratory tests and the diverse cadres of health workers needed to deliver the package of interventions necessary for effective HIV epidemic control. A participant opined as follows:

*‘You have to understand the scope of work needed for the national HIV response to be effective. It is daunting. Imagine all the necessary interventions and the funding required to meet all those needs. For instance, the health ministry says, ‘I want all the children living with HIV to receive a viral load test; we need to do contact tracing for every TB case you find. We need to identify new HIV positives in the community. Where will the resources come from? I don’t know whether there is an unlimited budget to implement all the HIV programming interventions. So, the donors have been pumping a lot of money into this HIV fight’* [District health team, Eastern Uganda,01]

Facility-level participants indicated that psycho-social support is integral to HIV care, which requires dedicated personnel such as ‘counsellors’ and ‘mentor mothers’ for pediatric clients to enhance adherence to ART. PWH frequently need sustained psycho-social support to adhere to treatment and to ensure that they are retained in care. All these combined efforts result in reduced HIV transmission at the community level. This has often necessitated community outreach that entails home visits for patient follow-up and adherence support. The operational costs of community outreach, which include health worker field monetary allowances, dedicated vehicle fleets, and attendant fuel budgets, were said to impinge on HIV care outside of the expenses for clinical care.

### Sub-national level HIV sector governance needs

Our case study reveals contrasts in donor funding arrangements across PEFAR and the SMGL project. Owing to stringent HIV epidemic control targets set by PEPFAR [], additional personnel are often recruited to augment the existing workforce at the sub-national level, such as HIV commodities supply chain experts. PEPFAR has its salary scales, several times the GoU salary scales. Salary disparities emerged as another barrier to the absorption of PEPFAR local hires. At the facility level, PEPFAR often provides salary support for data management personnel, counsellors, and select cadres not provided for in public sector staffing norms.

District health teams argued that the existing district health team set-up was not up to the task in meeting the demands of HIV epidemic control and planning in Eastern Uganda.

The latter participants conceded that they experienced governance deficits in effectively managing the multiple dimensions of HIV epidemic control after losing PEPFAR support. The current composition of district health teams was described as inadequate, with a weak capacity for effectively managing their governance mandate.

*‘In my office as the head of the district health team, you have the district health officer, a senior medical doctor with an MPH, then you have two assistants, one in charge of maternal care either a nurse or midwife, another for environmental health, then a senior health educator, who is part of environmental health then you have a biostatistician and that is it. Under heavens, you can never manage and supervise the interventions in the district, including managing this donor transition you are talking about* [District Health Team, Eastern Uganda,07]

Another perspective that emerged in our case study was that maternal and newborn care equipment, such as neonatal intensive care units (NICUs) were expensive to procure at the outset. However, these paid off after the initial financial investment. SMGL made substantial investments in expanding maternity wards, procuring ambulances and establishing advanced laboratories for district hospitals in Western Uganda. District health teams and facility in-charges argued that the initial financial investment bolstered supportive physical infrastructure development.

*‘We have had sustained gains in maternal and newborn care even after the end of USAID support because the heavy investments in infrastructure such as in setting up NICUs, buying ambulances, expanding maternity wards, all these combined investments are still giving returns’* [District Health Team, Western Uganda, 04].

On the other hand, the operational expenses incurred in HIV programming, such as community outreach activities for regular patient follow-up or contact tracing of PLHIV with active TB, are on a much more recurrent basis, requiring a constant cash flow.

*‘I think we shall still need external donors in the HIV fight for a long time in Uganda. HIV control is multi-faceted and needs lots of financial resources for HIV prevention within the population, for care and treatment, then you have these novel approaches like PrEP (pre-exposure prophylaxis), the vaginal ring (dapivirine) and the like, which require millions of dollars. I am not sure Uganda is prepared to set aside the kind of money needed to manage the HIV epidemic effectively* [Ministry of Health official, 05].

In terms of donor aid arrangements, HIV support by PEPFAR is more verticalized than maternal, newborn, and child health (MNCH). HIV services are frequently offered in parallel to general health services for those of GoU. There are disjointed HIV commodities supply chains, an additional layer of health workers and program managers recruited outside of the mainstream public service of Uganda, and sub-national stewardship arrangements divorced from the mainstream Ministry of Health governance set-up. These parallel structures were PEPFAR-funded and quickly unraveled in Eastern Uganda post-transition.

### Embeddedness of community outreach

The degree of embeddedness of community outreaches was a distinguishing feature between PEPFAR and SMGL support. The SMGL intervention tapped into existing village health teams (VHTs) and the existing lay community health workforce in villages across Uganda for community outreach activities. For instance, VHTs were co-opted in efforts to link women with pregnancy signs in the community with their first antenatal visit or to recommend those with complicated pregnancies to appropriate referral sites. The role of VHTs in linkage to facility-based maternal care in western Uganda beneficiary districts endured beyond SMGL project funding.

In the case of PEPFAR, community-linkage facilitators were recruited from among long-standing recipients of HIV care at facilities for patient follow-up in the community to ensure that they were retained in HIV care and not lost to follow-up. These were then compensated regularly with monetary allowances paid by PEPFAR through regionally based implementing organizations such as STAR-EC. This cadre of community health workers was not sustained after the PEPFAR transition. Facility-level informants reported that these were all lost after the PEPFAR transition because districts did not have a wage bill for retaining them post-transition, which contributed to increased cases of loss to follow-up.

*‘Community outreach activities ceased completely. We lost touch with our patients in the community. The linkage facilitators were salaried employees who were paid regularly for their role in patient follow-up deep in rural communities. When the allowances dried up, they could no longer continue. We lost these linkage facilitators after STAR-EC’s exit’* [Facility in-charge, Eastern Uganda, 10]

### Processes of transition

Our findings suggest differences in transition arrangements between PEPFAR and SMGL. Whereas both funders implemented transition arrangements for preparing beneficiaries for the end of project funding, there were nuanced differences in approach. Our comparison of the transition arrangements between the two funders as broadly informed by what Bennett and colleagues proposed as ‘best practices’ in donor transition [39]. There were noted differences in the categories of local stakeholders consulted, the level of engagement, for example, national, sub-national and facility-levels, actors’ seriousness in transition intention and perceived adequacy of communication around the transition from the perspective of project beneficiaries [39].

In the case of SMGL, district health teams and facility-level officials reported that they were informed right from the outset of the project that the SMGL intervention was a terminal one with no possibility of extension. District health teams and facility in-charges in western Uganda acknowledged attending multiple meetings. Baylor Uganda, the SMGL implementing organization, prepared districts for the end of the SMGL intervention at the start of the intervention in 2012. Participants indicated that the meetings were more frequent about two years towards the end of project funding. Overall, district health teams and facility in-charges in western Uganda expressed satisfaction with communication regarding the impending end of project funding made by Baylor Uganda.

*‘They kept reminding us about the end of the project and that we should prepare for it. I recall several meetings where they informed us of the looming end of the project. I think there was enough communication to us’* [Facility in-charge, Western Uganda,09]

Relating to districts in Eastern Uganda which lost PEPFAR support between 2015 and 2017, national-level actors such as Ministry of Health officials acknowledged that representatives of PEPFAR engaged them in several meetings discussing the impending loss of PEPFAR support in Luuka and Bulambuli districts. Ministry of Health officials indicated that they chaired some of the meetings called by PEPFAR to prepare them for loss of support in select districts.

Although representatives of PEPFAR engaged national-level actors such as Ministry of Health officials and other funders such as the Global Fund, these engagements were not held at sub-national level. District health teams and facility in-charges indicated they were not consulted about the impending loss of PEPFAR support and hence they did not adequately prepare for replacing lost PEPFAR investments in HIV programming. In terms of the participation of the varied actors, national-level officials were ‘informed’ rather than ‘consulted’ on the criteria or road map for transition. Sub-national level officials did not participate in these engagements.

*‘They informed us, but of course, the period was not so long. Everything happened within three months, informing us and transitioning, but there was no special training as such. They came here where we are seated, and they told us that now for Luuka (District), we are handing you over to the Ministry of Health to continue with HIV support’* [District Health Team, Eastern Uganda, 02]

Another contrasting feature in the transition arrangements was that unlike in the case of SMGL where project beneficiaries were made aware right from the onset that it was a terminal project, this was not the case for PEPFAR support. Indeed, it emerged from our interviews with officials at the national and sub-national levels that there was an unexpressed expectation that PEPFAR would resume support.

*‘We tried to plead with PEPFAR officials to return, but we tried to lobby, and they said ‘no’. We tried to argue that our district (Bulambuli) receives many visitors from neighboring districts. We told them that in this part of Uganda, we have mobile communities, we have a lot of population dynamics in HIV infection and if you just withdraw attention for just one year, HIV spreads but to no avail. Then in 2017, after STAR-EC (the outgoing PEPFAR IP) left, a successor IP was selected in RHITES EC. When a new IP came in, we had a lot of hope of PEPFAR’s return, but again, when RHITES came in, they also said ‘no’.* [District Health Team, Eastern Uganda, 02]

District health teams in Luuka and Bulambuli districts in Eastern Uganda indicated that PEPFAR resumed HIV programming support in their districts in October 2020 [22]. From their perspective, PEPFAR resumed support due to worsening indicators of HIV epidemic control in the districts [22]. The indicators cited by district health teams include HIV prevalence (such as in Luuka District, which was reportedly double that at pre-transition) [22]. Participants reported part of the source of data underpinning the policy reversal as data from the Uganda Population-based HIV Impact Survey (UPHIA) of 2016–2017 published in December 2020[29].

## Discussion

Our comparative case-study analysis suggests that key MNH indicators in western Uganda were perceived to have been sustained post-transition due to several facilitators. Participants identified the enablers that include timely follow-up external grants, a high rate of transition of SMGL-recruited health workers to the Government of Uganda (GoU) payroll and increased co-financing by GoU in MNH causes catalyzed by the SMGL intervention. On the other hand, significant barriers to the sustainment of HIV services in the post-transition dispensation in Eastern Uganda were identified by participants. These include the loss of a considerable proportion of the personnel previously hired by PEPFAR at national, sub-national and facility levels to accelerate progress towards attainment of HIV epidemic control targets, the apparent indifference by GoU in replacing lost PEPFAR investments in HIV programming, and the lack of alternative external assistance. The cessation of community outreach activities was perceived to have contributed to reversals in HIV epidemic control gains in case study districts in Eastern Uganda. A major contribution of our study is in illuminating drivers of health program sustainability outcomes across two health programs for which there has been little research previously [12,14,15].

### Health programming differences across HIV and MNH services

Our findings suggest that nuances in health programming needs across HIV and MNH programs are crucial in understanding variations in program sustainability outcomes. For instance, participants identified differences in health financing needs across the two health programs from a disease control or epidemiological perspective. District health teams in Eastern Uganda described HIV programming as ‘capital intensive’ and perceived domestic financing sources as inadequate in meeting the multi-faceted demands of HIV epidemic control at the population-level. From participants’ perspective, Uganda is heavily dependent on external donor aid for HIV programing relative to other health programs due to a range of reasons. These reasons include the high cost of HIV care and treatment, such as procurements of antiretrovirals, carrying out costly laboratory tests (such as viral load suppression), and the diverse cadres needed to manage the national HIV response at both national, sub-national and facility levels. Moreover, psycho-social support through regular visits to households of PWH is integral to HIV care, which requires dedicated personnel such as counsellors to promote adherence to ART. Yet, the operational costs of community outreach, including field monetary allowances for lay community health worker, were said to be daunting. Our observations concur with previous studies that have highlighted the challenges of financial sustainability of the global HIV response, given unique HIV epidemic control approaches [40,41].

Another notion that emerged in our case studies was that while the initial financial investment in maternal and newborn care equipment, such as neonatal intensive care units, was heavy, it continued giving returns even after the end of project funding by SMGL. On the other hand, the operational expenses incurred in HIV programming, such as community outreach, were perceived to be on a much more recurrent basis, hence requiring constant cash flow.

Accordingly, donor-funded HIV services were frequently offered parallel to those offered by GoU. There was an additional layer of health workers and program managers recruited outside of the mainstream public service of Uganda through a sub-national stewardship arrangement divorced from the mainstream governance systems of Ministry of Health. These parallel structures were PEPFAR-funded and quickly unraveled in Eastern Uganda post-transition. Scheirer and Dearing [26] have posited that not all interventions are created similarly; hence, implementation strategies may differ in structure and process. [42–44].

### Differences in global health funding priorities

Whereas MNH services in Western Uganda attracted multiple successor external funders such as the World Bank, Belgium’s ENABLE initiative, UNICEF and Save the Children, there was virtually no reported alternative external financing for HIV post-PEPFAR transition. Our case studies appear to suggest that at the time of data collection, MNH attracted more external assistance at the sub-national level in Uganda relative to HIV. Even at the level of domestic funding commitments, we observed that the GoU appeared more responsive to MNH in terms of funding commitments and transition responses. Further research is needed to understand the variability in external assistance commitment by health programs or conditions. Shiffman has written extensively on the political priority of MNH causes in LMICs, including on ‘the unacceptability of maternal death’ [44,45]. However, the idea that contrasting external actor interests drive global health financing is not entirely new [46–48].

### Donor transition ‘best practices’

Our case studies have implications for future donor transitions in health. We found that USAID’s SMGL project was perceived to have effectively communicated transition timelines in Western Uganda. Participants expressed satisfaction with the ample notice in alerting them to the end of project funding. This practice aligns with the Integrated Sustainability Framework [12], which amplifies the significance of transition processes, facilitating strategic planning for adaptation during post-transition periods to minimize disruptions in service delivery. On the other hand, local stakeholders in Uganda harbored expectations that PEPFAR would reverse course and resume aid. There is emerging evidence that around ‘responsible’ donor exits in health [49–51]. Burrows and colleagues [51] have proposed elaborate transition arrangements for donors and recipient governments to minimize disruptions in health service delivery occasioned by donor exits these include joint transition road maps, establishing clear timelines and transparent communication [39].

Although our study identifies differences in donor approaches across the two case-study projects, we note similarities in aid delivery mechanisms across USAID and PEPFAR. In both projects, aid was routed through international or local non-governmental agencies (e.g., STAR-EC for PEPFAR or Baylor-Uganda for USAID), and this aid was largely ‘off-budget’. Aid was time-limited, often in five-year grant cycles. Donor aid protocols and processes were determined globally rather than in-country. Support was usually for a set of countries in both cases, such as for PEPFAR focus countries and, in the case of SMGL, Uganda and Zambia were the beneficiary countries.

The implications are that donor transition decisions are often taken at an external level, such as in Washington D.C., with limited decision space for in-country actors, including on the donor side, to set agendas such as timelines or transition criteria.

Variations across the two projects emerged. We observe a more ‘vertical’ approach in PEPFAR’s support for the national HIV response in Uganda compared to USAID’s ‘Saving Mothers Giving Life’ project, which essentially went into strengthening the existing maternity services delivery infrastructure at district and sub-district hospitals. USAID’s SMGL initiative’s thrust was on ‘health systems strengthening’, such as in equipping theatres and enhancing workforce skills, which was not the principal aim of PEPFAR support. The differences in the sustainment of public health gains may indicate that DAH delivered via a ‘whole system approach’ may be more sustainable. Several studies have justified PEPFAR’s vertical approach as a necessity, particularly during the emergency phase of ART scale-up across LMICs due to weak health systems [42–44]. Additionally, we observe that a facilitator of health program sustainability was the absorption of SMGL workforce (such as nurses and midwives) onto the public sector payroll. It helped that this workforce was paid salaries comparable to their counterparts in public service. In contrast, sub-national level HIV program managers (such as supply chain experts) were not provided for in the public sector norms and were not absorbed post-PEPFAR transition. We observe that with respect to MNH, there was presence of internal champions who sourced for domestic resources to replace investment by SMGL which we did not notice with respect to districts which lost PEPFAR support. The literature on health program sustainability is consistent in identify

### Limitations

Our study had several limitations. A case study design such as that presented here is not intended to be broadly transferable to other settings: our aim was to provide an in-depth understanding of drivers of program sustainability of the two case study projects from the perspective of local stakeholders such as district health teams and frontline health workers in a range of different contexts in Uganda. While sub-national level HIV program managers perceived reversals in gains in HIV epidemic control between 2017 and 2020, the effects of Covid-19 lockdown on access to HIV services may not have been adequately accounted for.

## Conclusion

Our analysis suggests that variations in program sustainability outcomes across the two focus projects stem from differences in donor aid delivery mechanisms, transition processes, domestic political priorities, and nuances in health programming needs. Our study suggests that donor transition is not a ‘one size fits all’ phenomenon regarding health programs, which has implications for planning for donor transition in Uganda and similar settings.
